# Supplementary material for: Angelica sinensis polysaccharide as potential protectants against recurrent spontaneous abortion: focus on autophagy regulation
Source: Front Med (Lausanne). 2025 Jan 15;12:1522503. doi: 10.3389/fmed.2025.1522503 (PMC11774876; doi:10.3389/fmed.2025.1522503)
Supplement: Supplementary material S1 — The main instruments used during the LC-MS process, along with their models/specifications and manufacturers. [file Supplementary_file_1.zip › Metabolomics sequencing data FC1.2/8.代谢通路分析/Exposure_Ctrl/all/KEGG/mmu04371.html]

KEGG PATHWAY: Apelin signaling pathway - Mus musculus (house mouse)


# Apelin signaling pathway - Mus musculus (house mouse)


[
Pathway menu
| Organism menu
| Pathway entry
| Show description
| Download
| Help
]

Apelin is an endogenous peptide capable of binding the apelin receptor (APJ), which was originally described as an orphan G-protein-coupled receptor. Apelin and APJ are widely expressed in various tissues and organ systems. They are implicated in different key physiological processes such as angiogenesis, cardiovascular functions, cell proliferation and energy metabolism regulation. On the other hand, this ligand receptor couple is also involved in several pathologies including diabetes, obesity, cardiovascular disease and cancer.


##### Option

Scale:


100%

Image resolution:


 High

##### Search

##### ID search

##### Color


KGML

Image (png) file 1x
